# Association between weight-adjusted-waist index and cognitive decline in US elderly participants

**DOI:** 10.3389/fnut.2024.1390282

**Published:** 2024-06-06

**Authors:** Jiaxin Li, Jian Sun, Yishuo Zhang, Bo Zhang, Liya Zhou

**Affiliations:** Changchun University of Chinese Medicine, Changchun, China

**Keywords:** cognitive function, obesity, weight-adjusted-waist index, NHANES, cross-sectional study

## Abstract

**Objective:**

To investigate the association between the weight-adjusted-waist index (WWI) and cognitive decline in elderly Americans from 2011 to 2014.

**Methods:**

A cross-sectional study was conducted on 2,762 elderly participants from the National Health and Nutrition Examination (NHANES) between 2011 and 2014. WWI was calculated by dividing waist circumference (cm) by the square root of body weight (kg). Participants assessed their cognitive functions using tests such as the DSST, AFT, and CERAD W-L. In this research, multiple logistic regression, HIA, limited cubic spline (RCS), and threshold effect analysis methods were utilized to explore the relationship between cognitive decline and WWI.

**Results:**

The study involved 2,762 participants aged 60 years and older, comprising 1,353 males (49%) and 1,409 females (51%), with a median age of 69.3 years (standard deviation = 6.7). The analysis revealed that the risk of cognitive decline was positively associated with the WWI. Fully adjusted models indicated significant correlations with the CERAD W-L [odds ratio (OR) = 1.24, 95% confidence interval (CI) = 1.06–1.46, *p* < 0.008], AFT (OR = 1.27, 95% CI = 1.08–1.49, *p* = 0.003), and DSST (OR = 1.56, 95% CI = 1.29–1.9, *p* < 0.001). Subgroup analysis demonstrated a consistent relationship across different population settings except for gender (average of interactions, *p* > 0.05). A J-shaped relationship between WWI and low DSST scores was observed using multivariate restricted cubic spline (RCS) regression (*P* for non-linearity <0.05), with the curve steepening when WWI ≥ 12.21 cm/√kg. Additionally, the study found that WWI was more strongly associated with an increased risk of cognitive decline than other obesity indicators such as Body Mass Index (BMI), waist circumference (WC), and A Body Shape Index (ABSI).

**Conclusion:**

Our data have shown a significant positive association between the WWI and a higher risk of cognitive decline in older Americans, with a J-shaped non-linear relationship between WWI and DSST. In addition, our findings indicate that WWI was associated with greater cognitive decline than other markers of obesity.

## Introduction

As the global population ages, the deterioration of cognitive functions among the elderly is becoming an increasingly prominent issue. Senile cognitive decline, characterized by a progressive weakening of cognitive abilities including memory loss, inattention, and slower thought processes, is often seen in clinical conditions such as Alzheimer’s disease, vascular dementia, and mixed dementia. In 2015, approximately 46.8 million people were diagnosed with dementia globally, a number projected to rise to 130 million by 2050 (1). Cognitive decline significantly affects the elderly’s quality of life and social welfare, highlighting the importance of research and treatment for cognitive function decline in this demographic. The causes of cognitive decline in the elderly are complex, influenced by genetic, environmental, and lifestyle factors. While the role of genetics has been extensively explored, the effects of environmental and lifestyle factors are also being studied. Hence, managing these risk factors is crucial for slowing cognitive decline and preventing dementia.

Obesity is defined by an excessive accumulation of body fat, presenting significant health risks, and is often quantified using the Body Mass Index (BMI). Excessive nutrient intake can lead to insulin resistance (2), intestinal flora imbalance (3), oxidative stress, and other mechanisms (4), resulting in neuroinflammation and brain damage. As individuals age, their metabolic functions decline, increasing the likelihood of central obesity, a primary contributor to insulin resistance via oxidative stress (5). Central obesity is a key factor in inducing insulin resistance through oxidative stress. With higher levels of inflammatory cytokines (6), central obesity is more likely to cause brain structural abnormalities (7) and impact cognitive function. The weight-adjusted-waist index (WWI) (8) is a novel obesity index that primarily indicates central obesity by integrating the benefits of waist circumference (WC) and BMI (9). Currently, there is no definitive evidence connecting WWI with impaired cognitive function. We hypothesized a positive correlation between WWI levels and cognitive decline. To explore this, our study utilized data from the National Health and Nutrition Examination Survey (NHANES) to investigate the relationship between WWI and cognitive performance deterioration in older American adults. Additionally, we performed a comparative analysis to evaluate how traditional obesity indices like BMI, waist circumference, and A Body Shape Index (ABSI) relate to cognitive decline.

## Materials and methods

### Study population

**Figure 1:**
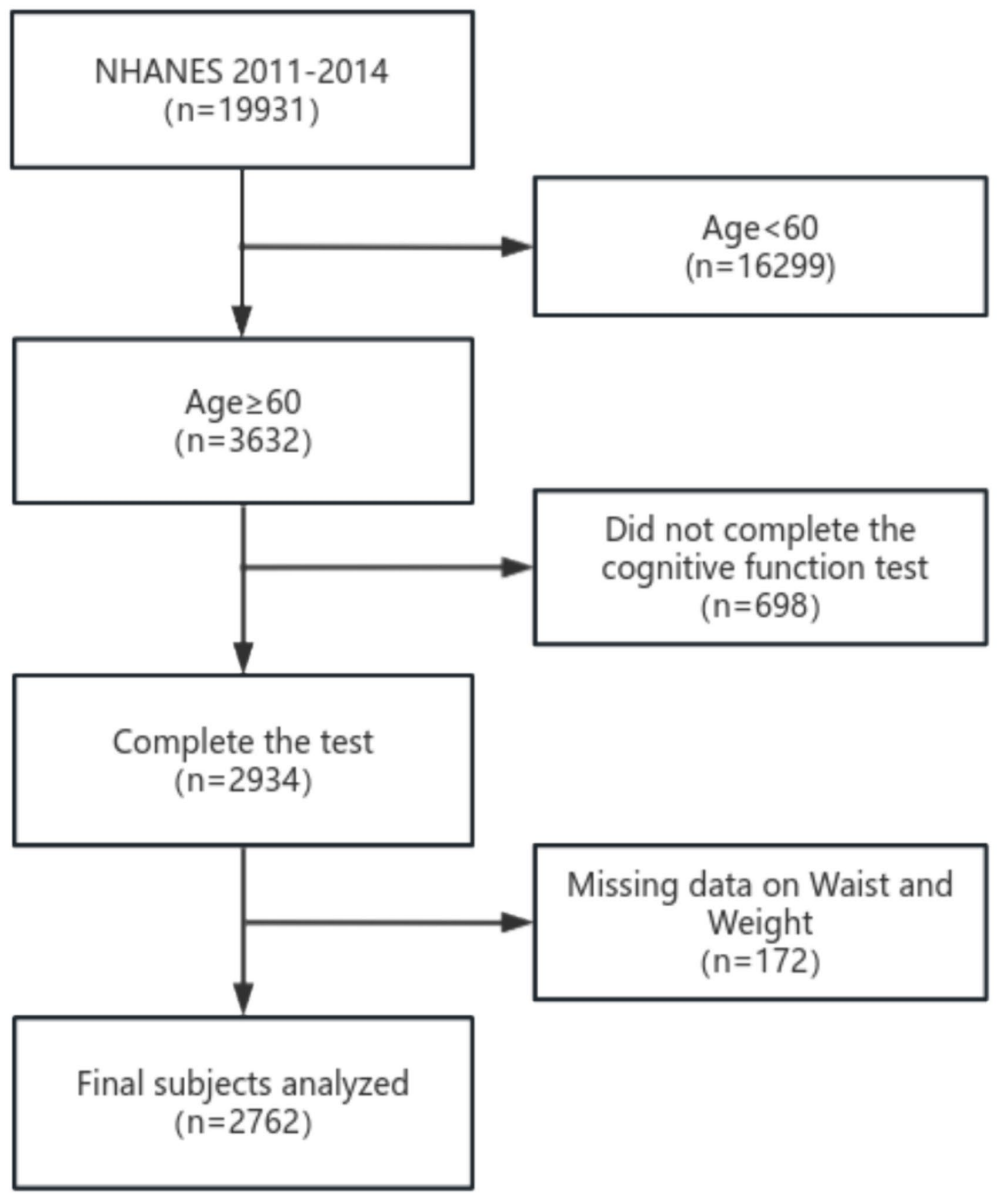
Flow chart of the screening process for the selection of eligible participants.

This study incorporated data from two cycles of the National Health and Nutrition Examination Survey (NHANES), part of a national program conducted by the National Center for Health Statistics (NCHS) to assess nutrition and health in the United States. The survey methodology was approved by the NCHS Research Ethics Review Committee, and all participants provided written informed consent. The study design and data are publicly accessible at www.cdc.gov/nchs/nhanes/. Cognitive function data were collected during the 2011–2012 and 2013–2014 NHANES cycles, involving a total of 19,931 individuals. Participants under 60 years old, those with incomplete cognitive data, or missing WWI measurements were excluded, leaving 2,762 eligible participants for analysis (Figure 1).

### Measurement of WWI

WWI (cm/√kg) was calculated by dividing weight (kg) by the square root of waist (cm). Health technicians, who were trained in body measurement techniques, assessed waist circumference and weight at a mobile examination center (MEC). Information on waist circumference and weight was made publicly available on the NHANES website through the “Body Measurement” feature.


$$WWI=\frac{\text{WC}\left(\text{cm}\right)}{\sqrt{\text{weight}\left(\text{kg}\right)}}$$

Additionally, we investigated the correlation between obesity indices, including waist circumference (WC), body mass index (BMI), A Body Shape Index (ABSI) and cognitive function decline. ABSI was determined using the formula WC/(BMI^2/3^ × height^1/2^). In order to avoid ABSI has a large regression coefficient, we multiplied this value by 1,000.

### Cognitive function

A battery of assessments was used to gauge cognitive function in the mobile testing center (MEC), which included the Alzheimer’s Disease Registry Consortium (CERAD) word learning sub-test, the Animal Fluency Test (AFT), and the Digit Symbol Substitution Test (DSST). These tests specifically assess executive function, linguistic skills, working memory, and processing speed in the elderly. For example, the CERAD test involves three initial learning trials and one delayed recall trial. Participants are required to read aloud 10 unrelated words individually and then recall as many words as possible immediately after. The AFT and DSST are conducted prior to the delayed recall of the CERAD test. The final score for the CERAD test is the sum of the three learning trials and the delayed recall, each scored from 0 to 10. The Animal Fluency Test measures language fluency by asking participants to name as many animals as they can in 1 minute, with each correct name scoring one point. The DSST tasks participants with matching numbers to symbols within a set time to evaluate processing speed, sustained attention, and working memory. Currently, there is no specific threshold set for low cognitive performance on the CERAD, AFT, or DSST tests. For this study, we adopted the 25th percentile (lowest quartile) of scores as the cutoff point, following the precedent set by existing research and aligning with methodologies described in the literature (10–12).

### Covariates

We included a range of covariates previously identified in the literature as related to obesity factors and cognitive function. These primarily included social and economic variables (e.g., age), health-related behavior variables (e.g., drinking status), and health-related variables (e.g., diabetes). Detailed descriptions and classifications of these covariates are provided in Table 1.

**Table 1:** The classifications of covariates.

Covariates	Classification
Gender	Male; female
Age(year)	60–70; 70–80; ≥80
Race	Mexican American; Other Hispanic; Non-Hispanic White; Non-Hispanic Black; Non-Hispanic Asian; Other Race
Educational Level	Less than high school; High school or GED; Above high school
Marital status	Married; Widowed; Divorced; Separated; Never married; Living with partner
Poverty income ratio	≥1; ≤0.99
Drinking Status	<12 drinks/year; ≥12 drinks/year
Smoking Status	Current; Former; Never
Body mass index	<25 kg/m^2^; 25-30 kg/m^2^; ≥30 kg/m^2^
Diabetes	No; yes
Hypertension	No; yes
Coronary Heart Disease	No; yes
Hyperlipidemic	No; yes
Stroke	No; yes
Chronic Bronchitis	No; yes

### Statistical analysis

We employed the Kolmogorov–Smirnov test to ascertain the normal distribution of variables. Variables with a normal distribution are presented as mean ± *SD*, while those with a skewed distribution are expressed as median (interquartile range [IQR]). Categorical variables are reported as frequency (%). To examine differences among various WWI groups, we employed one-way analysis of variance for normally distributed data, Kruskal-Wallis H tests for skewed data, and Chi-square or Fisher’s exact tests for categorical data. A binary logistic regression model (showing odds ratios [OR] and 95% confidence intervals [CI]) was used to evaluate the influence of WWI on cognitive decline, adjusting for key covariates. WWI was analyzed as a continuous variable. The choice of these confounders was informed by existing scientific literature (11, 13, 14). Three models were developed: Model 1 without any covariate adjustments; Model 2 adjusted for age, sex, race, education level, marital status, and poverty-to-income ratios; and Model 3 further adjusted for BMI, diabetes, hyperlipidemia, hypertension, stroke, coronary heart disease, and chronic bronchitis. To assess trends and explore potential nonlinearity, WWI was divided into categorical quintiles. A generalized additive model (GAM) with natural splines (Section 4) was used to explore the nonlinear relationship between WWI and cognitive decline by including a quadratic term in the regression model. If a nonlinear relationship was indicated, a two-piecewise regression model was planned to identify the threshold effect of WWI on cognitive function based on smooth plot visualization. Effect sizes and *p*-values from these models were documented and compared. The subgroup analysis was conducted using the following variables: Gender, age (60–69 vs. 70–79 or ≥ 80 years), BMI (<25 vs. 25–30 or ≥ 30 kg/m^2^) and history of relevant diseases. A multi-variable logical regression model is used to evaluate the heterogeneity of subgroups, and the interaction is tested in comparison. All analyses were performed using Version 1.9 of the Free Statistics analysis platform, located in Beijing, China, and R Statistical Software (version 4.2.2, http://www.R-project.org, The R Foundation). A two-sided *p* value <0.05 was considered statistically significant.

## Results

### Baseline characteristics

After implementing inclusion and exclusion criteria, the study involved 2,762 participants, comprising 1,353 (49%) males and 1,409 (51%) females, with a median age of 69.3 years (*standard deviation* = 6.7). The ethnic composition included 9% Mexican American, 10.4% other Hispanic, 23.6% non-Hispanic Black, 47.2% non-Hispanic White, 8.4% non-Hispanic Asian, and 1.4% from other racial groups. The mean values of the cognitive function tests were as follows: CERAD W-L 25.0 (6.4), AFT 16.7 (5.5), and DSST 46.3 (17.1). Table 2 provides a detailed view of the participants’ clinical characteristics, with columns displaying the number of quintile divisions based on WWI. Compared to the lowest quintile, individuals in the highest quintile of WWI tend to be older, have a higher proportion of women, include more Mexican American, other Hispanic, and non-Hispanic White individuals, and have a larger number of widowed and divorced participants. Additionally, this group shows a significantly higher prevalence of diabetes, hypertension, coronary heart disease, dyslipidemia, stroke, and chronic bronchitis.

**Table 2:** Characteristics of the study population, National Health and Nutrition Examination Survey (NHANES) 2011–2014 (*N* = 2,762).

Characteristic	Weight-adjusted-waist index						
	Total	Q1 (<10.89)	Q2 (10.89–11.31)	Q3 (11.31–11.65)	Q4 (11.65–12.06)	Q5 (>12.06)	*p*-value
NO.	2,762	553	552	552	552	553	
Age(year),Mean ± *SD*	69.3 ± 6.7	67.1 ± 6.2	69.0 ± 6.6	69.2 ± 6.6	70.2 ± 6.8	70.9 ± 6.8	< 0.001
Gender, *n* (%)							< 0.001
Male	1,353 (49.0)	330 (59.7)	282 (51.1)	308 (55.8)	253 (45.8)	180 (32.5)	
Female	1,409 (51.0)	223 (40.3)	270 (48.9)	244 (44.2)	299 (54.2)	373 (67.5)	
Race, *n* (%)							< 0.001
Mexican American	249 (9.0)	32 (5.8)	37 (6.7)	55 (10)	65 (11.8)	60 (10.8)	
Other Hispanic	288 (10.4)	27 (4.9)	69 (12.5)	61 (11.1)	56 (10.1)	75 (13.6)	
Non-Hispanic White	1,304 (47.2)	218 (39.4)	243 (44)	254 (46)	289 (52.4)	300 (54.2)	
Non-Hispanic Black	651 (23.6)	204 (36.9)	150 (27.2)	124 (22.5)	97 (17.6)	76 (13.7)	
Non-Hispanic Asian	232 (8.4)	65 (11.8)	45 (8.2)	48 (8.7)	43 (7.8)	31 (5.6)	
Other Race	38 (1.4)	7 (1.3)	8 (1.4)	10 (1.8)	2 (0.4)	11 (2)	
Educational Level, *n* (%)							< 0.001
Less than high school	694 (25.1)	113 (20.4)	122 (22.1)	135 (24.5)	144 (26.1)	180 (32.5)	
High school or GED	644 (23.3)	108 (19.5)	133 (24.1)	128 (23.2)	131 (23.7)	144 (26)	
Above high school	1,422 (51.5)	332 (60)	296 (53.6)	289 (52.4)	277 (50.2)	228 (41.2)	
Marital status, *n* (%)							< 0.001
Married	1,542 (55.8)	337 (60.9)	327 (59.2)	335 (60.7)	290 (52.5)	253 (45.8)	
Widowed	512 (18.5)	63 (11.4)	87 (15.8)	85 (15.4)	114 (20.7)	163 (29.5)	
Divorced	394 (14.3)	73 (13.2)	76 (13.8)	72 (13)	91 (16.5)	82 (14.8)	
Separated	78 (2.8)	23 (4.2)	20 (3.6)	12 (2.2)	9 (1.6)	14 (2.5)	
Never married	158 (5.7)	40 (7.2)	27 (4.9)	25 (4.5)	37 (6.7)	29 (5.2)	
Living with partner	78 (2.8)	17 (3.1)	15 (2.7)	23 (4.2)	11 (2)	12 (2.2)	
Poverty income ratio, % (SE)							< 0.001
≥1	2,108 (83.4)	443 (87.2)	443 (87.4)	416 (83.9)	426 (82.7)	380 (75.5)	
≤0.99	421 (16.6)	65 (12.8)	64 (12.6)	80 (16.1)	89 (17.3)	123 (24.5)	
Drinking Status, % (SE)							< 0.001
<12drinks/year	854 (31.2)	145 (26.5)	167 (30.5)	149 (27.2)	176 (32.1)	217 (39.7)	
≥12drinks/year	1881 (68.8)	402 (73.5)	380 (69.5)	398 (72.8)	372 (67.9)	329 (60.3)	
Smoking Status							0.131
Current	296 (21.1)	71 (25.7)	52 (17.4)	63 (22.3)	51 (18.9)	59 (21.4)	
Former	57 (4.1)	12 (4.3)	18 (6)	11 (3.9)	6 (2.2)	10 (3.6)	
Never	1,049 (74.8)	193 (69.9)	228 (76.5)	208 (73.8)	213 (78.9)	207 (75)	
Body mass index, % (SE)							< 0.001
<25 kg/m^2^	729 (26.5)	268 (48.7)	168 (30.5)	133 (24.1)	95 (17.3)	65 (11.8)	
25-30 kg/m^2^	972 (35.3)	198 (36)	213 (38.7)	230 (41.7)	183 (33.3)	148 (26.9)	
≥30 kg/m^2^	1,051 (38.2)	84 (15.3)	169 (30.7)	188 (34.1)	272 (49.5)	338 (61.3)	
WC, Mean ± *SD*	101.9 ± 14.7	90.1 ± 10.6	97.4 ± 11.4	101.8 ± 11.5	106.7 ± 13.2	113.4 ± 14.7	< 0.001
ABSI, Mean ± *SD*	52.8 ± 9.7	55.0 ± 9.2	53.3 ± 9.2	53.1 ± 9.0	52.1 ± 10.2	50.7 ± 10.1	< 0.001
Diabetes, % (SE)	635 (23.0)	60 (10.8)	101 (18.3)	122 (22.1)	151 (27.4)	201 (36.3)	< 0.001
Hypertension, % (SE)	1704 (61.7)	276 (49.9)	333 (60.3)	337 (61.1)	368 (66.7)	390 (70.5)	< 0.001
Coronary Heart Disease, % (SE)	243 (8.8)	30 (5.4)	43 (7.8)	51 (9.2)	43 (7.8)	76 (13.7)	< 0.001
Hyperlipidemic, % (SE)	1,555 (56.3)	246 (44.5)	308 (55.8)	330 (59.8)	338 (61.2)	333 (60.2)	< 0.001
Stroke, % (SE)	177 (6.4)	30 (5.4)	31 (5.6)	31 (5.6)	32 (5.8)	53 (9.6)	0.02
Chronic Bronchitis, % (SE)	196 (7.1)	23 (4.2)	29 (5.3)	30 (5.4)	56 (10.1)	58 (10.5)	< 0.001
CERAD W-L Test, Mean ± *SD*	25.0 ± 6.4	25.9 ± 6.4	25.1 ± 6.5	24.9 ± 6.3	24.8 ± 6.4	24.4 ± 6.6	0.002
Animal Fluency Test, Mean ± *SD*	16.7 ± 5.5	17.1 ± 5.7	16.9 ± 5.7	17.0 ± 5.2	16.6 ± 5.4	15.9 ± 5.3	0.001
DSST,Mean ± *SD*	46.3 ± 17.1	49.0 ± 17.4	47.1 ± 16.4	47.3 ± 17.0	46.0 ± 17.0	42.0 ± 16.9	< 0.001

### Relationship between weight-adjusted-waist index and cognitive decline

WWI was a continuous variable positively associated with the likelihood of cognitive decline in univariate logistic regression analyses (CERAD W-L OR = 1.28, 95%CI = 1.14–1.44, *p* < 0.001; AFT OR = 1.23, 95%CI = 1.1–1.38, *p* < 0.001; DSST OR = 1.46, 95%CI = 1.29–1.66, *p*< 0.001) (Table 3). The relationship between WWI and cognitive function remained significant even after adjusting for age and sex. Further adjustments revealed that each one standard deviation (SD) increase in WWI increased the risk of scoring low on the CERAD W-L by 28% (95% CI = 1.06–1.46, *p* < 0.008), increased the risk of scoring low on the AFT by 27% (95% CI = 1.08–1.49, *p* = 0.003), and increased the risk of scoring low on the DSST by 56% (95% CI = 1.29–1.9, *p* < 0.001) (Table 3, Model 3).

**Table 3:** Association between WWI and cognitive decline.

Variable	Model 1		Model 2		Model 3	
	OR (95%CI)	*p-*value	OR (95%CI)	*P*-value	OR (95%CI)	*p*-value
CERAD W-L Test	1.28 (1.14–1.44)	<0.001	1.15 (0.99–1.32)	<0.001	1.24 (1.06–1.46)	<0.008
1st Quintile (<10.89)	Ref		Ref		Ref	
2st Quintile (10.89–11.31)	1.35 (1.03–1.77)	0.031	1.18 (0.86–1.61)	0.297	1.25 (0.91–1.72)	0.17
3st Quintile (11.31–11.65)	1.37 (1.05–1.8)	0.022	1.11 (0.81–1.52)	0.516	1.2 (0.87–1.66)	0.271
4st Quintile (11.65–12.06)	1.42 (1.08–1.86)	0.011	1.12 (0.81–1.53)	0.494	1.27 (0.9–1.77)	0.169
5st Quintile (>12.06)	1.64 (1.25–2.14)	<0.001	1.27 (0.92–1.75)	0.153	1.45 (1.01–2.07)	0.043
p for trend		0.001		0.277		0.079
AFT	1.23 (1.1–1.38)	<0.001	1.19 (1.03–1.37)	0.015	1.27(1.08–1.49)	0.003
1st Quintile (<10.89)	Ref		Ref		Ref	
2st Quintile (10.89–11.31)	1.01 (0.78–1.31)	0.932	0.99 (0.74–1.34)	0.973	1.04 (0.76–1.42)	0.802
3st Quintile (11.31–11.65)	0.93 (0.72–1.21)	0.602	0.89 (0.65–1.21)	0.447	0.95 (0.69–1.31)	0.749
4st Quintile (11.65–12.06)	1 (0.77–1.3)	0.985	0.95 (0.7–1.29)	0.744	1.04 (0.75–1.45)	0.808
5st Quintile (>12.06)	1.43 (1.11–1.85)	0.006	1.37 (1–1.87)	0.047	1.52 (1.08–2.14)	0.017
p for trend		0.012		0.091	1.09 (1.01–1.18)	0.033
DSST	1.46 (1.29–1.66)	<0.001	1.41 (1.19–1.68)	<0.001	1.56 (1.29–1.9)	<0.001
1st Quintile (<10.89)	Ref		Ref		Ref	
2st Quintile (10.89–11.31)	1.06 (0.79–1.41)	0.7	0.93 (0.64–1.35)	0.702	0.95 (0.65–1.4)	0.79
3st Quintile (11.31–11.65)	1.12 (0.84–1.49)	0.454	0.95 (0.65–1.38)	0.781	1.04 (0.7–1.54)	0.854
4st Quintile (11.65–12.06)	1.3 (0.98–1.72)	0.07	1.14 (0.79–1.66)	0.48	1.29 (0.87–1.94)	0.208
5st Quintile (>12.06)	1.97 (1.5–2.59)	<0.001	1.7 (1.17–2.47)	0.006	1.94 (1.27–2.96)	0.002
p for trend		<0.001		0.002		<0.001

Q, quintiles; OR, odd ratio; CI, confidence interval; Ref: reference. Model 1 was unadjusted. Model 2 was adjusted for Age, Gender, Race, Educational level, Marital status, and Poverty income ratio. Model 3 was adjusted for Model 2 + BMI, Diabetes, Hypertension, Hyperlipidemia, Coronary Heart Disease, Stroke, and Chronic Bronchitis. CERAD W-L Test¸AFT¸DSST scores were converted into categorical variable.

When WWI was divided into five alleles, individuals in the highest group (Q5) showed a significantly increased likelihood of experiencing cognitive decline by 45, 52, and 94% higher compared to those in the lowest quintile, for CERAD W-L (OR = 1.45, 95% CI = 1.01–1.07, *p* = 0.043), AFT (OR = 1.52, 95% CI = 1.08–2.14, *p* = 0.017), and DSST (OR = 1.94, 95% CI = 1.27–2.96, *p* = 0.002), respectively, as reported in Table 3, Model 3.

**Figure 2:**
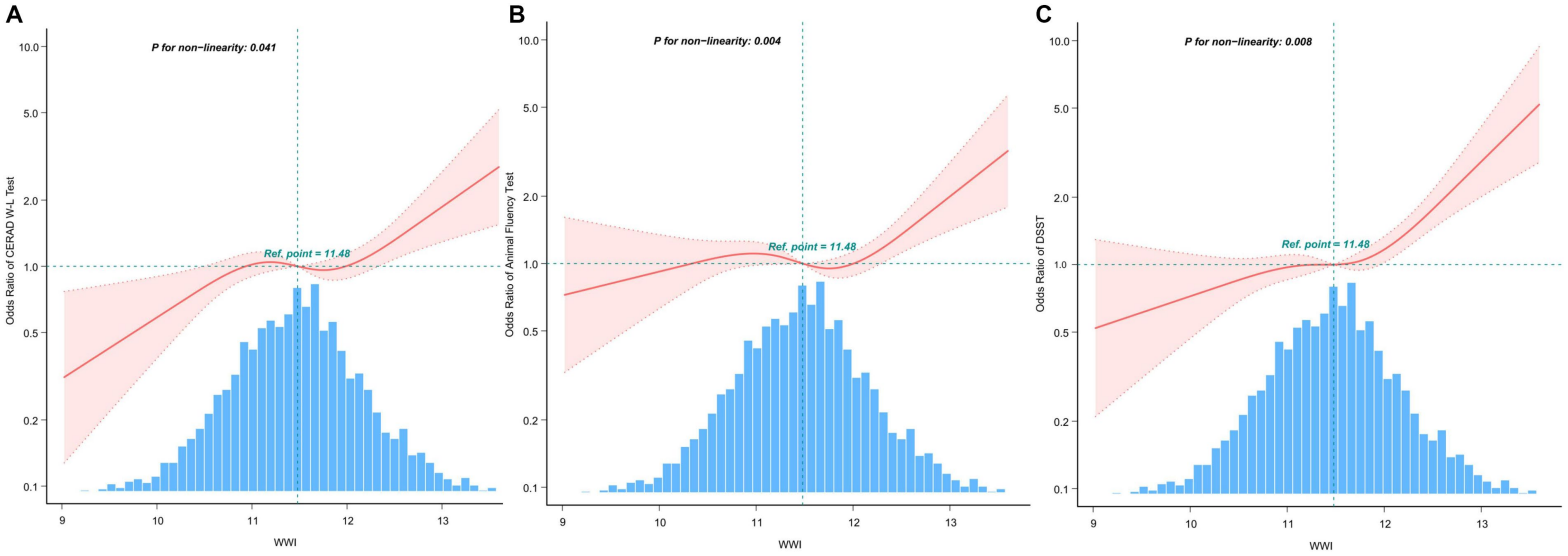
After covariate adjustment, restricted cubic spline plots for the outcome of cognitive deterioration by WWI levels. **(A)** CERAD W-L Test. **(B)** AFT. **(C)** DSST. The backdrop histograms, which are colored light blue, show the percentage of the research population’s WWI density distribution. The computed adjusted odds ratios are shown by heavy center lines, while the 95% confidence intervals are shown by shaded ribbons. The odds ratio of 1.0 is shown by the horizontal dotted lines (Reference point).

The multivariate-adjusted limited cubic spline analysis revealed a J-shaped curve depicting the relationship between WWI and lower scores on CERAD W-L, AFT, and DSST tests (CERAD W-L *P* for nonlinearity<0.05, AFT *P* for nonlinearity = 0.004, DSST *P* for nonlinearity = 0.008) as shown in Figure 2. This analysis indicated a nonlinear dose–response connection, with the prevalence of cognitive impairment increasing as WWI increases In threshold analysis, when WWI was less than 12.21 cm/√kg, the odds ratio of low DSST was 1.256 (95%CI: 1.043–1.512, *p* = 0.016); when WWI is ≥12.21 cm/√kg, the odds ratio of low DSST was 1.966 (95%CI: 1.014–3.81, *p* = 0.0453), both of which had statistical significance. The population with WWI ≥ 12.21 cm/√kg accounted for 14.81% of the total population and 73.96% of the top quintile population. When the threshold was 12.716 cm/√kg, the OR value of low CERAD W-L was 1.186 (95%CI: 1.024–1.373, *p* = 0.00226), which was statistically significant. However, this significant relationship disappeared at WWI ≥ 12.716 cm/√kg (Table 4).

**Table 4:** Threshold effect analysis of relationship of WWI with cognitive decline.

	Total, % (*n* = 2,762)	Q5, % (*n* = 553)	Adjusted model	
			OR(95% CI)	*p*-value
**CERAD W-L Test**				
<12.716	2,635 (95.6%)	426 (77.03%)	1.186 (1.024–1.373)	0.00226
≥12.716	127 (4.5%)	127 (22.97%)	1.946 (0.425–8.91)	0.3909
Likelihood Ratio test			–	0.587
**AFT**				
<12.367	2,468 (89.36%)	259 (46.84%)	1.022 (0.874–1.195)	0.7832
≥12.367	294 (10.64%)	294 (53.16%)	1.633 (0.696–3.834)	0.2599
Likelihood Ratio test			–	0.138
**DSST**				
<12.21	2,352(85.19%)	144 (26.04%)	1.256 (1.043–1.512)	0.016
≥12.21	409(14.81%)	409 (73.96%)	1.966 (1.014–3.81)	0.0453
Likelihood Ratio test			–	0.187

OR, odds ratio; CI, confidence interval. Adjusted for Age and Gender. 99.8% of the data is shown.

### Stratified analyses based on additional variables

**Figure 3:**
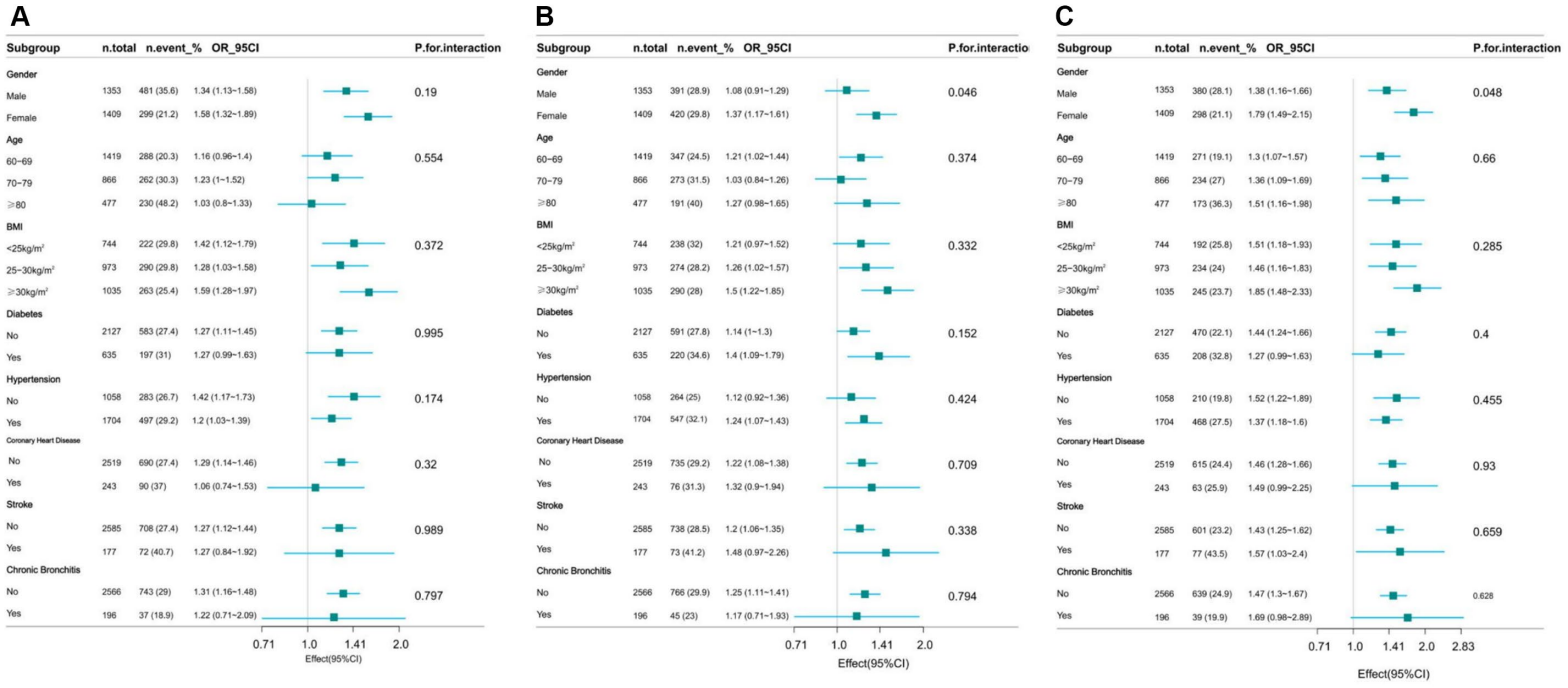
Effect of WWI on cognitive decline in different subgroup (gender, age, health factors). **(A)** CERAD W-L Test. **(B)** AFT. **(C)** DSST.

The study performed stratified analyses across different subgroups to evaluate whether the relationship between WWI and cognitive decline varied among these groups. After stratification by factors such as gender, age, BMI, and history of relevant diseases, the results showed no significant interactions in any of the subgroups except gender (AFT *p* = 0.046, DSST *p* = 0.048), with all interaction *p*-values exceeding 0.05 (Figure 3).

### The weight-adjusted-waist index exhibited a stronger correlation with cognitive decline compared to other obesity markers (BMI, WC, and ABSI)

**Figure 4:**
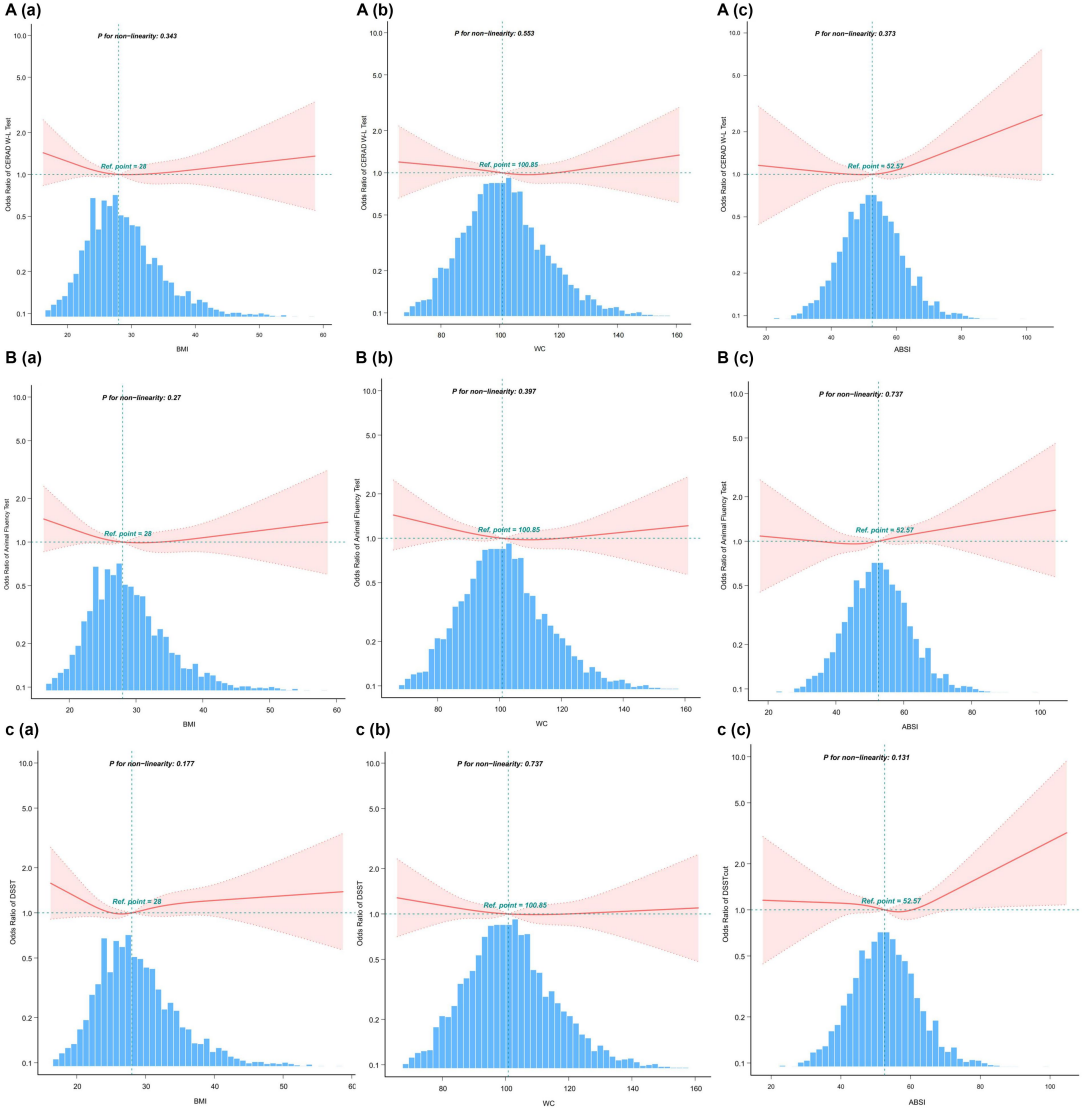
Restricted cubic spline plots for Cognitive decline outcome by BMI, WC and ABSI levels after covariate adjustment. **(A)** CERAD W-L Test. **(B)** AFT. **(C)** DSST. The background histograms (light blue color) represent the per cent of the density distribution of WWI in the study population. Heavy central lines represent the estimated adjusted odds ratios, with shaded ribbons denoting 95% confidence intervals. The horizontal dotted lines represent the odds ratio of 1.0 (Reference point).

The indicators of obesity, such as waist circumference (WC), body mass index (BMI), and a body shape index (ABSI), and their association with cognitive decline, were analyzed using smooth curve fitting, as illustrated in Figure 4. A segmented regression model was applied to define the intervals, and threshold effects were calculated, with the results presented in Table 5.

**Table 5:** Threshold effect analysis of relationship of BMI, WC and ABSI with cognitive decline.

	BMI	WC	ABSI
Breakpoint (K) (CERAD W-L Test)	30.905	29.43	64.771
OR1 (<K)	0.988 (0.957–1.021)	0.996 (0.987–1.005)	1.006 (0.994–1.019)
	0.4733	0.3733	0.3377
OR2 (≥K)	1.023 (0.988–1.06)	1.01 (0.984–1.038)	1.089 (1.032–1.149)
	0.1957	0.4471	0.002
Likelihood Ratio test	0.201	0.331	0.008
Breakpoint (K) (AFT)	116.856	103.146	45.264
OR1 (<K)	0.989 (0.971–1.007)	0.99 (0.976–1.004)	1.002 (0.956–1.05)
	0.2246	0.1497	0.9388
OR2 (≥K)	1.068 (0.983–1.16)	1.004 (0.992–1.017)	1.013 (1–1.025)
	0.1217	0.5042	0.0463
Likelihood Ratio test	0.078	0.215	0.744
Breakpoint (K) (DSST)	77.504	75.971	62.915
OR1 (<K)	1.005 (0.989–1.022)	0.987 (0.943–1.034)	0.991 (0.978–1.005)
	0.546	0.5908	0.2173
OR2(≥K)	0.927 (0.643–1.338)	0.999 (0.991–1.007)	1.024 (0.98–1.07)
	0.6861	0.7741	0.2885
Likelihood Ratio test	0.542	0.611	0.221

OR, odds ratio; CI, confidence interval. Adjusted for Age and Gender. 99.8% of the data is shown.

Additionally, we compared the odds ratio (OR) values of WWI with those of other obesity indicators, calculated their z-scores, and utilized them for a multifactor analysis (Table 6). The findings showed that WWI was more strongly associated with poor cognitive function than other obesity markers such as WC, BMI, and ABSI. Specifically, the OR values were as following: CERAD W-L, WWI: OR = 1.24, BMI: OR = 1, WC: OR = 1, ABSI: OR = 1.01; AFT, WWI:OR = 1.27, BMI: OR = 0.99, WC: OR = 1, ABSI: OR = 1.01; DSST, WWI: OR = 1.56, BMI: OR = 0.98, WC: OR = 0.99, ABSI: OR = 1.02. These results suggest that WWI may be a more effective predictor of low cognitive function than other commonly used obesity markers.

**Table 6:** Association between WWI,BMI,WC,ABSI and cognitive decline.

	WWI	BMI	WC	ABSI
z-scores	OR (P5%CI), *p*-value	OR (P5%CI), *p*-value	OR (P5%CI), *p*-value	OR (P5%CI), *p*-value
Continuous (CERAD W-L)	1.24 (1.06–1.46), *p* < 0.008	1 (0.98–1.01), *p* = 0.699	1 (0.99–1.01), *p* = 0.924	1.01 (1–1.02), *p* = 0.092
Continuous (AFT)	1.27(1.08–1.49), *p* = 0.003	0.99 (0.97–1), *p* = 1.116	1 (0.99–1), *p* = 0.537	1.01 (1–1.02), *p* = 0.014
Continuous (DSST)	1.56 (1.29–1.9), *p* < 0.001	0.98 (0.96–1), *p* = 0.078	0.99 (0.99–1), *p* = 0.204	1.02 (1.01–1.03), *p* = 0.003

OR, odds ratio; CI, confidence interval. Adjusted for Age, Gender, Race, Educational level, Marital status, Poverty income ratio, Diabetes, Hypertension, Hyperlipidemia, Coronary Heart Disease, Stroke, and Chronic Bronchitis.

## Discussion

In this extensive cross-sectional study using NHANES data from 2011 to 2014, it was discovered that the weight-adjusted waist index (WWI) might be significantly linked to cognitive function decline, displaying a J-shaped non-linear relationship (*P* for nonlinearity <0.05). Notably, the relationship curve becomes steeper at WWI levels exceeding 12.21 cm/√kg, especially in the DSST test. No interaction was observed between WWI and cognitive decline, indicating consistent findings across different subgroups. By placing the WWI quintile, the low cognitive impairment test was significantly more associated with WWI (> 12.06 cm/√kg) than the lowest WWI category (< 10.89 cm/√kg). Additionally, our results showed that WWI had a stronger correlation with reduced cognitive function compared to other obesity indices, suggesting that WWI could be a more effective predictor of cognitive decline than other measures related to obesity. Obesity has been shown to diminish cognitive function across the lifespan (15). Interestingly, several prior studies have confirmed the correlation between obesity indicators such as BMI and WC and impaired cognitive function. Momtaz et al. (16) conducted a cohort study on the elderly population in Malaysia and discovered a significant correlation between low cognitive performance and BMI. Similarly, Huang et al. (17) identified a positive correlation between higher BMI and cognitive decline in younger individuals. Benito-Leon et al. (18) reported that individuals who were overweight or obese scored lower on cognitive function tests, a finding supported by related Mendelian randomization studies (19, 20). In addition to BMI, studies have also revealed a potential relationship between hippocampal volume, waist-to-hip ratio, and visceral obesity, with waist-to-hip ratio being positively associated with high white matter signal intensity (21, 22). Obesity in older adults may result in reduced metabolic activity in the frontal lobe and anterior cingulate gyrus, as well as shrinkage of the hippocampus and thalamus, impacting cognitive functions (23–28). A meta-analysis conducted by Tang et al. (7), which included 21 studies and 2,060,687 participants, found that high WC is a risk factor for developing cognitive impairments, underscoring the role of central obesity in cognitive decline. While BMI and WC are commonly employed to assess obesity, they are not without their flaws, and there are ongoing discussions about the “obesity paradox” (29, 30). Consequently, traditional obesity indicators like WC and BMI may not fully capture the role of central obesity in predicting cognitive impairment. WWI, as a newer standard for measuring obesity, not only minimizes the association with BMI but also incorporates WC. This allows for a more precise and comprehensive assessment of central obesity, thereby providing a clearer depiction of the relationship between obesity and cognitive function. Recent studies have demonstrated effectiveness of WWI in differentiating muscle mass from fat mass, leading to its broader application in various medical domains including metabolic diseases, kidney diseases, and cardiovascular diseases, among others (31–33). Furthermore, WWI has shown a greater correlation and predictive power in some diseases compared to traditional obesity indices (34, 35). In our study, WWI was found to have a much stronger correlation with poor cognitive function than obesity markers such as BMI, WC, and ABSI (CERAD W-L, WWI: 1.24, BMI: 1, WC: 1, ABSI: 1.01; AFT, WWI: 1.27, BMI: 0.99, WC: 1, ABSI: 1.01; DSST, WWI: 1.56, BMI: 0.98, WC: 0.99, ABSI: 1.02). These findings further support the potential of WWI as a predictor of obesity-related diseases.

Cognitive function tests in the NHANES survey comprised the CERAD test, the animal fluency test, and the number symbol substitution test. These tests evaluated immediate and delayed learning ability, verbal fluency, and executive and attention skills, respectively. Existing research indicates that different factors impact various functional areas of the brain, leading to diverse outcomes across these tests. However, our study identified a significant correlation between the WWI and lower scores on all three cognitive function tests, suggesting that WWI has a comprehensive influence on cognitive decline. While only a limited number of previous studies have investigated the relationship between WWI and cognitive performance, our results align with these studies, confirming that WWI is positively associated with cognitive decline. However, our study adds new insights, by identifying a nonlinear positive correlation and a J-shaped dose–response relationship in the DSST scores, evident in both unadjusted and adjusted models. We also refined the inclusion of covariates by considering detailed past medical histories, including diabetes, hypertension, hyperlipidemia, history of stroke, coronary heart disease, and chronic bronchitis. This approach highlights how obesity is connected not only to metabolic and respiratory disorders but also to increased risks of cardiovascular and cerebrovascular diseases (36). In addition, some studies have found that cognitive dysfunction that cognitive dysfunction might begin in the initial stages of chronic airway damage and worsen further as the disease deteriorates (37), but relevant evidence is still needed to confirm it. Our study has many advantages. Including the use of a nationally representative sample of elderly individuals from the National Survey on the Health and Nutritional Status of the Elderly. For the first time, we compared the effects of the WWI on cognitive decline against traditional obesity indicators, affirming superiority of WWI. Moreover, various confounding factors were also meticulously accounted for to accurately assess the relationship between WWI and cognitive function. However, our study is not without its limitations. Firstly, to better delineate the potential risk relationship between WWI and low cognitive function during statistical analysis, we categorized the lowest four quintiles of test scores as indicative of low cognitive function, transforming continuous variables into categorical ones. Secondly, the cross-sectional design of our study limits our ability to establish a causal relationship between WWI and cognitive decline. This limitation is a primary focus for our next phase of research, where we aim to corroborate the universality of this relationship through more comprehensive and clinically robust data from multiple countries. Additionally, despite our rigorous consideration of numerous significant confounding factors, we cannot completely rule out the influence of unmeasured confounders related to the risk of cognitive decline. Thus, future studies should aim to address these limitations to validate and expand upon our findings.

## Conclusion

In elderly individuals in the United States, an increase in the WWI was associated with an increased risk of cognitive decline. Furthermore, the relationship between WWI and cognitive decline proved stronger than with other obesity indices, indicating that WWI could serve as a reliable indicator of cognitive decline.

## Data availability statement

The datasets presented in this study can be found in online repositories: https://www.cdc.gov/nchs/nhanes/index.htm, further inquiries can be directed to the corresponding author.

## Ethics statement

The studies involving humans were approved by National Center for Health Statistics Research Ethics Review Board. The studies were conducted in accordance with the local legislation and institutional requirements. The participants provided their written informed consent to participate in this study. Written informed consent was obtained from the individual(s) for the publication of any potentially identifiable images or data included in this article.

## Author contributions

JL: Conceptualization, Data curation, Writing – original draft, Writing – review & editing. JS: Conceptualization, Methodology, Writing – original draft. YZ: Formal analysis, Software, Supervision, Writing – review & editing. BZ: Formal analysis, Software, Supervision, Writing – review & editing. LZ: Formal analysis, Funding acquisition, Writing – review & editing.

## References

[1] Prince M Ali G-C Guerchet M Prina AM Albanese E Wu Y-T. Recent global trends in the prevalence and incidence of dementia, and survival with dementia. Alzheimers Res Ther. (2016) 8:23. doi: 10.1186/s13195-016-0188-8, PMID: 27473681 PMC4967299

[2] Kothari V Luo Y Tornabene T O’Neill AM Greene MW Geetha T. High fat diet induces brain insulin resistance and cognitive impairment in mice. Biochim Biophys Acta Mol basis Dis. (2017) 1863:499–508. doi: 10.1016/j.bbadis.2016.10.006 27771511

[3] Saiyasit N Chunchai T Prus D Suparan K Pittayapong P Apaijai N. Gut dysbiosis develops before metabolic disturbance and cognitive decline in high-fat diet–induced obese condition. Nutrition. (2020) 69:110576. doi: 10.1016/j.nut.2019.110576, PMID: 31580986

[4] Hajiluian G Abbasalizad Farhangi M Nameni G Shahabi P Megari-Abbasi M. Oxidative stress-induced cognitive impairment in obesity can be reversed by vitamin D administration in rats. Nutr Neurosci. (2017) 21:744–52. doi: 10.1080/1028415x.2017.1348436, PMID: 28683595

[5] Barzilai N Huffman DM Muzumdar RH Bartke A. The critical role of metabolic pathways in aging. Diabetes. (2012) 61:1315–22. doi: 10.2337/db11-1300, PMID: 22618766 PMC3357299

[6] Santos AL Sinha S. Obesity and aging: molecular mechanisms and therapeutic approaches. Ageing Res Rev. (2021) 67:101268. doi: 10.1016/j.arr.2021.101268 33556548

[7] Tang X Zhao W Lu M Zhang X Zhang P Xin Z. Relationship between central obesity and the incidence of cognitive impairment and dementia from cohort studies involving 5,060,687 participants. Neurosci Biobehav Rev. (2021) 130:301–13. doi: 10.1016/j.neubiorev.2021.08.028, PMID: 34464646

[8] Park Y Kim NH Kwon TY Kim SG. A novel adiposity index as an integrated predictor of cardiometabolic disease morbidity and mortality. Sci Rep. (2018) 8:16753. doi: 10.1038/s41598-018-35073-4, PMID: 30425288 PMC6233180

[9] Qin Z Chang K Yang Q Yu Q Liao R Su B. The association between weight-adjusted-waist index and increased urinary albumin excretion in adults: a population-based study. Front Nutr. (2022) 9:941926. doi: 10.3389/fnut.2022.941926, PMID: 36034904 PMC9412203

[10] Gong Z Song W Gu M Zhou X Tian C. Association between serum iron concentrations and cognitive impairment in older adults aged 60 years and older: a dose-response analysis of National Health and nutrition examination survey. PLoS One. (2021) 16:e0255595. doi: 10.1371/journal.pone.0255595, PMID: 34339453 PMC8328322

[11] Dong X Li S Sun J Li Y Zhang D. Association of Coffee, decaffeinated coffee and caffeine intake from coffee with cognitive performance in older adults: National Health and nutrition examination survey (NHANES) 2011–2014. Nutrients. (2020) 12:840. doi: 10.3390/nu12030840, PMID: 32245123 PMC7146118

[12] Peeri NC Egan KM Chai W Tao M-H. Association of magnesium intake and vitamin D status with cognitive function in older adults: an analysis of US National Health and nutrition examination survey (NHANES) 2011 to 2014. Eur J Nutr. (2020) 60:465–74. doi: 10.1007/s00394-020-02267-4, PMID: 32388734 PMC7649128

[13] Wang X Wang R Zhang Z Luo C Zhao Z Ruan J. Level-specific associations of urinary antimony with cognitive function in US older adults from the National Health and nutrition examination survey 2011–2014. BMC Geriatr. (2022) 22:663–11. doi: 10.1186/s12877-022-03351-6, PMID: 35962346 PMC9375424

[14] Wang A Zhao M Luo J Zhang T Zhang D. Association of Dietary Vitamin K Intake with Cognition in the elderly. Front Nutr. (2022) 9:900887. doi: 10.3389/fnut.2022.900887, PMID: 35811956 PMC9260313

[15] Wang C Chan JSY Ren L Yan JH. Obesity reduces cognitive and motor functions across the lifespan. Neural Plast. (2016) 2016:1–13. doi: 10.1155/2016/2473081, PMID: 26881095 PMC4737453

[16] Momtaz YA Haron SA Hamid TA Ibrahim R Tanjani PT. Body mass index (BMI) and cognitive functions in later life. Curr Alzheimer Res. (2018) 15:195–200. doi: 10.2174/1567205014666171004114246 28982334

[17] Huang T Chen Z Shen L Fan X Wang K. Associations of cognitive function with BMI, body fat mass and visceral fat in young adulthood. Medicina. (2019) 55:221. doi: 10.3390/medicina55060221, PMID: 31142005 PMC6631832

[18] Benito-León J Mitchell AJ Hernández-Gallego J Bermejo-Pareja F. Obesity and impaired cognitive functioning in the elderly: a population-based cross-sectional study (NEDICES). Eur J Neurol. (2013) 20:899. doi: 10.1111/ene.12083, PMID: 23323838

[19] Marioni RE Yang J Dykiert D Mõttus R Campbell A Davies G. Assessing the genetic overlap between BMI and cognitive function. Mol Psychiatry. (2016) 21:1477–82. doi: 10.1038/mp.2015.205, PMID: 26857597 PMC4863955

[20] Mina T Yew YW Ng HK Sadhu N Wansaicheong G Dalan R. Adiposity impacts cognitive function in Asian populations: an epidemiological and Mendelian randomization study. Lancet Reg Health West Pac. (2023) 33:100710. doi: 10.1016/j.lanwpc.2023.100710, PMID: 36851942 PMC9957736

[21] Isaac V Sim S Zheng H Zagorodnov V Tai ES Chee M. Adverse associations between visceral adiposity, brain structure, and cognitive performance in healthy elderly. Front Aging Neurosci. (2011) 3:12. doi: 10.3389/fnagi.2011.00012, PMID: 21949507 PMC3171695

[22] Jagust W Harvey D Mungas D Haan M. Central obesity and the aging brain. Arch Neurol. (2005) 62:1545–8. doi: 10.1001/archneur.62.10.1545 16216937

[23] Ho A Raji C Parikshak N Becker J Lopez O Kuller L. Brain structure and obesity. NeuroImage. (2009) 47:S109. doi: 10.1016/s1053-8119(09)70976-0 PMC2826530 19662657

[24] Bolzenius JD Laidlaw DH Cabeen RP Conturo TE McMichael AR Lane EM. Brain structure and cognitive correlates of body mass index in healthy older adults. Behav Brain Res. (2015) 278:342–7. doi: 10.1016/j.bbr.2014.10.010, PMID: 25448431 PMC4382378

[25] Taki Y Kinomura S Sato K Inoue K Goto R Okada K. Relationship between body mass index and gray matter volume in 1,428 healthy individuals. Obesity. (2008) 16:119–24. doi: 10.1038/oby.2007.4, PMID: 18223623

[26] Volkow ND Wang G Telang F Fowler JS Goldstein RZ Alia-Klein N. Inverse association between BMI and prefrontal metabolic activity in healthy adults. Obesity. (2009) 17:60–5. doi: 10.1038/oby.2008.469, PMID: 18948965 PMC2681079

[27] Walther K Birdsill AC Glisky EL Ryan L. Structural brain differences and cognitive functioning related to body mass index in older females. Hum Brain Mapp. (2010) 31:1052–64. doi: 10.1002/hbm.20916, PMID: 19998366 PMC6870943

[28] Willette AA Kapogiannis D. Does the brain shrink as the waist expands? Ageing Res Rev. (2015) 20:86–97. doi: 10.1016/j.arr.2014.03.007, PMID: 24768742 PMC4538938

[29] Geng J Deng L Qiu S Bian H Cai B Li Y. Low lean mass and cognitive performance: data from the National Health and nutrition examination surveys. Aging Clin Exp Res. (2021) 33:2737–45. doi: 10.1007/s40520-021-01835-w, PMID: 33786800

[30] Dinu M Colombini B Pagliai G Vannetti F Pasquini G Molino Lova R. BMI, functional and cognitive status in a cohort of nonagenarians: results from the Mugello study. Eur Geriatr Med. (2020) 12:379–86. doi: 10.1007/s41999-020-00417-9, PMID: 33085046 PMC7990833

[31] Yu S Wang B Guo X Li G Yang H Sun Y. Weight-adjusted-waist index predicts newly diagnosed diabetes in Chinese rural adults. J Clin Med. (2023) 12:1620. doi: 10.3390/jcm12041620, PMID: 36836156 PMC9961347

[32] Li X Wang L Zhou H Xu H. Association between weight-adjusted-waist index and chronic kidney disease: a cross-sectional study. BMC Nephrol. (2023) 24:266. doi: 10.1186/s12882-023-03316-w 37691097 PMC10494374

[33] Fang H Xie F Li K Li M Wu Y. Association between weight-adjusted-waist index and risk of cardiovascular diseases in United States adults: a cross-sectional study. BMC Cardiovasc Disord. (2023) 23:435. doi: 10.1186/s12872-023-03452-z, PMID: 37658325 PMC10474739

[34] Wen Z Li X. Association between weight-adjusted-waist index and female infertility: a population-based study. Front Endocrinol. (2023) 14:1175394. doi: 10.3389/fendo.2023.1175394, PMID: 37614708 PMC10442810

[35] Zhang D Shi W Ding Z Park J Wu S Zhang J. Association between weight-adjusted-waist index and heart failure: Results from National Health and nutrition examination survey 1999–2018. Front Cardiovasc Med. (2022) 9:1069146. doi: 10.3389/fcvm.2022.1069146, PMID: 36588556 PMC9794568

[36] Khudiakova AD Polonskaya YV Shramko VS Shcherbakova LV Striukova EV Kashtanova EV. Blood Adipokines/cytokines in young people with chronic bronchitis and abdominal obesity. Biomol Ther. (2022) 12:1502. doi: 10.3390/biom12101502, PMID: 36291711 PMC9599484

[37] Dal Negro R Bonadiman L Tognella S Briccolo F Turco P. Extent and prevalence of cognitive dysfunction in chronic obstructive pulmonary disease, chronic non-obstructive bronchitis, and in asymptomatic smokers, compared to normal reference values. Int J Chron Obstruct Pulmon Dis. (2014) 9:675–83. doi: 10.2147/copd.s63485, PMID: 25061286 PMC4085326

