# Supplementary material for: Association between weight-adjusted-waist index and cognitive decline in US elderly participants
Source: Front Nutr. 2024 Jun 6;11:1390282. doi: 10.3389/fnut.2024.1390282 (PMC11187255; doi:10.3389/fnut.2024.1390282)
Supplement: Supplementary file 1 [file Table_1.docx]

**Supplementary Table 1** **Association between different variables and Cognitive decline.**

| **Variable** | **CERAD W-L Test** | | **Animal Fluency Test** | | **DSST** | |
| --- | --- | --- | --- | --- | --- | --- |
|  | **OR, 95%CI** | ***P*-Value** | **OR, 95%CI** | ***P*-Value** | **OR, 95%CI** | ***P*-Value** |
| **Age** |  |  |  |  |  |  |
| 60-69 | Ref |  | Ref |  | Ref |  |
| 79-79 | 1.74 (1.44~2.11) | <0.001 | 1.44 (1.19~1.74) | <0.001 | 1.63 (1.34~1.99) | <0.001 |
| ≥80 | 3.58 (2.85~4.49) | <0.001 | 2.02 (1.61~2.54) | <0.001 | 2.37 (1.88~3) | <0.001 |
| **Gender** |  |  |  |  |  |  |
| Male | Ref |  | Ref |  | Ref |  |
| Female | 0.49 (0.41~0.58) | <0.001 | 1.04 (0.89~1.23) | 0.6 | 0.69 (0.58~0.82) | <0.001 |
| **Race** |  |  |  |  |  |  |
| Mexican American | Ref |  | Ref |  | Ref |  |
| Other Hispanic | 1.08 (0.76~1.54) | 0.653 | 1.42 (0.98~2.05) | 0.065 | 1.38 (0.98~1.95) | 0.068 |
| Non-Hispanic White | 0.67 (0.5~0.89) | 0.006 | 0.66 (0.49~0.9) | 0.009 | 0.27 (0.2~0.37) | <0.001 |
| Non-Hispanic Black | 0.72 (0.52~0.98) | 0.037 | 1.96 (1.42~2.7) | <0.001 | 0.92 (0.68~1.24) | 0.572 |
| Non-Hispanic Asian | 0.54 (0.36~0.81) | 0.003 | 1.88 (1.28~2.75) | 0.001 | 0.31 (0.2~0.48) | <0.001 |
| Other Race | 0.67 (0.31-1.43) | 0.298 | 1.08 (0.51-2.31) | 0.833 | 0.31 (0.13-0.78) | 0.013 |
| **Educational Level** |  |  |  |  |  |  |
| Less than high school | Ref |  | Ref |  | Ref |  |
| High school or GED | 0.45 (0.36-0.57) | <0.001 | 0.58 (0.47-0.73) | <0.001 | 0.23 (0.18-0.29) | <0.001 |
| Above high school | 0.28 (0.23-0.35) | <0.001 | 0.28 (0.23-0.34) | <0.001 | 0.08 (0.06-0.1) | <0.001 |
| **Marital status** |  |  |  |  |  |  |
| Married | Ref |  | Ref |  | Ref |  |
| Widowed | 1.4 (1.13-1.74) | 0.002 | 1.62 (1.31-2) | <0.001 | 2.03 (1.63-2.52) | <0.001 |
| Divorced | 0.75 (0.58-0.98) | 0.034 | 0.89 (0.69-1.14) | 0.358 | 1.02 (0.77-1.33) | 0.914 |
| Separated | 1.33 (0.82-2.15) | 0.252 | 1.91 (1.21-3.04) | 0.006 | 4 (2.53-6.35) | <0.001 |
| Never married | 1.02 (0.71-1.48) | 0.897 | 0.89 (0.61-1.29) | 0.524 | 1.25 (0.85-1.83) | 0.258 |
| Living with partner | 1.18 (0.72-1.93) | 0.512 | 0.62 (0.35-1.1) | 0.105 | 1.06 (0.61-1.84) | 0.836 |
| **Poverty income ratio** |  |  |  |  |  |  |
| ≥1 | Ref |  | Ref |  | Ref |  |
| ≤0.99 | 1.64 (1.31-2.04) | <0.001 | 2.09 (1.68-2.59) | <0.001 | 3.17 (2.55-3.95) | <0.001 |
| **Diabetes** |  |  |  |  |  |  |
| No | Ref |  | Ref |  | Ref |  |
| Yes | 1.19 (0.98-1.45) | 0.076 | 1.38 (1.14-1.66) | 0.001 | 1.72 (1.41-2.09) | <0.001 |
| **Hypertension** |  |  |  |  |  |  |
| No | Ref |  | Ref |  | Ref |  |
| Yes | 1.13 (0.95-1.34) | 0.17 | 1.42 (1.2-1.69) | <0.001 | 1.53 (1.27-1.84) | <0.001 |
| **Hyperlipidemic** |  |  |  |  |  |  |
| No | Ref |  | Ref |  | Ref |  |
| Yes | 0.85 (0.72-1) | 0.049 | 0.89 (0.75-1.05) | 0.162 | 0.92 (0.77-1.09) | 0.34 |
| **Coronary Heart Disease** |  |  |  |  |  |  |
| No | Ref |  | Ref |  | Ref |  |
| Yes | 1.56 (1.18-2.05) | 0.002 | 1.1 (0.83-1.47) | 0.493 | 1.08 (0.8-1.46) | 0.601 |
| **Stroke** |  |  |  |  |  |  |
| No | Ref |  | Ref |  | Ref |  |
| Yes | 1.82 (1.33-2.48) | <0.001 | 1.76 (1.29-2.4) | <0.001 | 2.54 (1.86-3.47) | <0.001 |
| **Chronic Bronchitis** |  |  |  |  |  |  |
| No | Ref |  | Ref |  | Ref |  |
| Yes | 0.57 (0.4-0.82) | 0.003 | 0.7 (0.5-0.99) | 0.042 | 0.75 (0.52-1.08) | 0.118 |
| **BMI** |  |  |  |  |  |  |
| <25kg/m2 | Ref |  | Ref |  | Ref |  |
| 25-30kg/m2 | 1 (0.81-1.23) | 0.988 | 0.83 (0.68-1.03) | 0.086 | 0.91 (0.73-1.13) | 0.404 |
| ≥30kg/m2 | 0.8 (0.65-0.99) | 0.039 | 0.83 (0.67-1.02) | 0.071 | 0.89 (0.72-1.11) | 0.302 |
| **WWI** | 1.28 (1.14-1.44) | <0.001 | 1.23 (1.1-1.38) | <0.001 | 1.46 (1.29-1.66) | <0.001 |
| **WC** | 1 (1-1.01) | 0.71 | 1 (0.99-1) | 0.1 | 1 (0.99-1) | 0.689 |
| **ABSI(*1000)** | 1.01 (1-1.01) | <0.001 | 1 (1-1) | 0.007 | 1 (1-1.01) | <0.001 |

OR,odds ratio；CI,confidence interval.
